# What is the future for surgical oncology?

**DOI:** 10.1038/bjc.2011.447

**Published:** 2011-11-22

**Authors:** A M Thompson

**Affiliations:** 1 Department of Surgical Oncology, University of Dundee, Ninewells Hospital and Medical School, Dundee DD1 9SY, UK

Clinical cancer research in the United Kingdom has undoubtedly strengthened in the past decade (Stead *et al*, 2011) and with future multidisciplinary support and if funding from government and non-governmental agencies is sustained should continue to thrive. However, the role of individual specialities, such as surgical oncology, has been questioned (Glynn and Sweeney, 2011) reflecting the current reappraisal of surgical research more generally (McCulloch, 2011).

After a century of surgical oncology, surgical research into technical innovations and randomised trials of surgical approaches, while still necessary, in the field of cancer has been largely replaced by surgical input into multicentre trials and clinical studies. This builds on the proud tradition of international surgical leadership in trials of prevention, screening, neoadjuvant and adjuvant therapies, continuing in current trials. Even in the metastatic setting, prospective, surgically led studies can demonstrate the value of surgical biopsy of recurrent disease influencing therapy for one in six patients and informing future trial protocols (Thompson *et al*, 2010).

However, surgical oncologists do need to maintain both intellectual and craft capabilities, which may displace the time others spend on collaborative research and clinical trials. The tendency of surgical specialists to congregate in the relative comfort of their own societies, rather than become involved in wider cancer communities, may reinforce a perception of isolation. The current need for increasing documentation (ethics, NHS Research and Development, local paper trails) where simple local solutions were once possible and the insistence that Good Clinical Practice training is needed for everyone contributing to clinical trials inevitably present obstacles. In combination, such factors act to hinder surgical input into collaborative research. However, these hurdles can be overcome. For example, surgeons currently chair several multidisciplinary UK national cancer bodies such as the Clinical Trial Awards Advisory Committee funding clinical trials in the UK and key site-specific National Cancer Research Network Clinical Studies Groups.

The future for surgical oncology is indeed to collaborate but also to integrate with other specialties in multidisciplinary, cancer site-specific teams addressing the issues of the day. Surgeons with oncological practices need to bring their personal drive, dedication and intellectual rigour to the cancer trials and research teams. By integrating into local, regional, national and international organisations, surgical oncologists will continue to enhance the quality and success of cancer trials and studies that change clinical practice and benefit individual patients and populations.
